# Salfonal—A New Hypnotic

**Published:** 1888-08

**Authors:** 


### SULFONAL—A NEW HYPNOTIC.

Synthetic chemistry has, in recent years, added many valuable organic compounds to our materia medica.

The latest candidate for medical endorsement is a modest little compound, which hides its blushes behind the somewhat cumbersome name of "Diethylsulfondimethylmethan."

Professor Kast, of Freiburg, has considerately abbreviated this to "Sulfonal." This high German authority claims to have discovered in Sulfonal what we have so long looked for, namely, a safe hypnotic. By the word *safe* is meant one which is absolutely free from deleterious after-effects, and which does not enslave, as do other narcotics and hypnotics. Its administration, in doses of from fifteen to sixty grains, is said to be followed by natural and refreshing sleep, lasting from five to eight hours. From this no unpleasant after-effects are seen; the patient feels as much invigorated as from normal sleep, and what is better than this, there is no tendency whatever to form a "habit."

The drug has already been used by Dr. Rabbas (Berliner Klinische Wochenschrift, No. 17, 1888) with great success in over two hundred cases of insomnia. We hope all that has been said about it is true, but it is prudent to hold back our enthusiasm until other observers have handed in their reports.

If we mistake not, all this, and more, was said about chloral when it was first introduced to the profession. We believe it is not yet for sale in the American market.

